# Effect of pulse laser frequency on PLD growth of LuFeO_3_ explained by kinetic simulations of in-situ diffracted intensities

**DOI:** 10.1038/s41598-022-09414-3

**Published:** 2022-04-05

**Authors:** Vít Gabriel, Pavel Kocán, Sondes Bauer, Berkin Nergis, Adriana Rodrigues, Lukáš Horák, Xiaowei Jin, Reinhard Schneider, Tilo Baumbach, Václav Holý

**Affiliations:** 1 Department of Surface and Plasma Science, Charles University, V Holešovičkách 2, 180 00 Prague 8, Czech Republic; 2 Institute for Photon Science and Synchrotron Radiation, Karlsruhe Institute of Technology, Hermann-von-Helmholtz-Platz 1, 76344 Eggenstein-Leopoldshafen, Germany; 3 Department of Condensed Matter Physics, Charles University, Ke Karlovu 5, 121 16 Prague 2, Czech Republic; 4 Laboratory for Electron Microscopy, Karlsruhe Institute of Technology, Engesserstr. 7, 76131 Karlsruhe, Germany; 5 Department of Condensed Matter Physics, Masaryk University, Kotlářská 2, 611 37 Brno, Czech Republic

**Keywords:** Surfaces, interfaces and thin films, Atomistic models

## Abstract

Atomistic processes during pulsed-laser deposition (PLD) growth influence the physical properties of the resulting films. We investigated the PLD of epitaxial layers of hexagonal LuFeO$_3$ by measuring the X-ray diffraction intensity in the quasiforbidden reflection 0003 in situ during deposition. From measured X-ray diffraction intensities we determined coverages of each layer and studied their time evolution which is described by scaling exponent $\beta$ directly connected to the surface roughness. Subsequently we modelled the growth using kinetic Monte Carlo simulations. While the experimentally obtained scaling exponent $\beta$ decreases with the laser frequency, the simulations provided the opposite behaviour. We demonstrate that the increase of the surface temperature caused by impinging ablated particles satisfactorily explains the recorded decrease in the scaling exponent with the laser frequency. This phenomena is often overlooked during the PLD growth.

## Introduction

Multiferroic materials have attracted a lot of attention because of their unique physical properties. Hexagonal LuFeO$_3$ is a prototypical example of a single-phase multiferroic compound, since it exhibits simultaneously ferroelectric and magnetic ordering at room temperature^1–4^. For the epitaxial growth of single-crystalline thin films of LuFeO$_3$ and other Lu–Fe–O phases pulsed-laser deposition (PLD) is frequently used^4–7^. The structure quality of resulting layers, especially the interface sharpness is often comparable to molecular-beam epitaxy, however the PLD method still requires a deep understanding of the growth mechanism at the atomistic scale.

In addition to varied microscopic methods devoted to the structure study of PLD-grown layers after the growth completion, such as atomic-force microscopy (AFM), transmission and scanning electron microscopy (TEM, SEM), as well as scanning tunneling microscopy (STM), in-situ X-ray scattering has been employed in a row of works, see the reviews in Refs.^5,8,9^. The evolution of the mesoscopic surface morphology during PLD can be investigated in situ by grazing incidence small-angle X-ray scattering (GISAXS), which reveals the growth of monolayer islands, terrace movement, and the development of surface roughness during deposition^10–12^. Standard X-ray diffraction (XRD) and reciprocal-space mapping investigates the time dependence of the crystalline quality and the lattice mosaicity in particular^7^.

A true atomic resolution is achieved by surface X-ray diffraction (SXRD), which measures the distribution of scattered intensity in reciprocal space along truncation rods perpendicular to the crystal surface. The intensity in so-called anti-Bragg maxima (AB) between ordinary diffraction maxima ($00\frac{1}{2}$ for instance) is extremely sensitive to the surface structure. This approach was used in several works for various material types such as semiconductors^13^, metals^14^, organic layers^15,16^, and complex oxides^4,5,8,17–21^. The AB intensity is sensitive to partially occupied monolayers so that it is suitable especially for the investigation of the layer-by-layer (LBL) growth mode^22^. Kowarik et al. showed^23^ that kinetics of surface roughening can also be investigated by intensity measurement in several points $00q, q=\frac{1}{2},\frac{2}{3},\frac{3}{4},\ldots$ on the crystal truncation rod.

The analysis of the kinetics of the LBL growth from the AB intensity is based on a suitable growth model. The most popular model formulated by Cohen et al.^24^ considers the time evolution of the coverages $\theta _j(t)$ of individual monolayers, which covers deposition of adatoms on individual terraces, attachment of the adatoms to steps, and adatom movement across the steps. A modification of the model was published in Ref.^15^, where the authors considered also the Ehrlich-Schwoebel diffusion barrier (ES) for the interlayer transport. Extensive numerical simulations in these works demonstrated that the individual coverage profiles can be approximated by a hyperbolic tangent function^13^

1 $$\begin{aligned} \theta _j(t) \approx [1+\tanh ((t-t_j)/w_j)]/2, \end{aligned}$$

where $t_j$ is the time at which the *j*-th monolayer is half-filled, and $w_j$ is the characteristic width of the *j*-th profile, the growth rate is defined as $r_j=1/(t_j-t_{j-1})$. We also define the relative profile width $p_j=w_jr_j$. Braun et al. in the cited paper compared the AB intensities measured during MBE homoepitaxy of GaAs and found a power scaling law of the widths $w_j \sim t^\beta$. In the classical work on dynamical scaling^25^ the coefficient $\beta$ was introduced as the *growth exponent* describing the dependence of the interface width on deposition time. The growth exponent strongly depends on the growth temperature. In case of high temperature the deposited particles have enough energy to recrystallize to lower-energy state, which is the flat layer with minimum of steps. Corresponding growth approaches the perfect LBL, profile widths do not change and $\beta = 0$. In case of low temperatures there is no relaxation and the crystal grows randomly without inter-layer transport. In this case each subsequent profile is wider than its predecessor because it takes longer time to fill next layers and $\beta$ will be maximum.

In this paper we firstly investigate the PLD growth of hexagonal LuFeO$_3$ epitaxial layers by measuring AB X-ray diffraction in situ during the growth. For the analysis of the measured data, we utilised a kinetic Monte Carlo (kMC) model ^26^. A study of the $\beta$ exponent dependency on the laser frequency was performed for both measured and simulated data. The measured dependence of the growth exponent on the laser frequency *f* was justified by a raising in the substrate temperature upon increasing *f*.

## Results

### Experimental results

**Figure 1:**
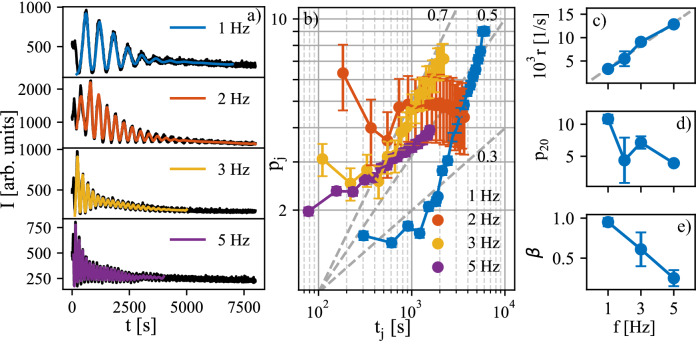
(**a**) Measured AB intensities (points) of samples deposited with various laser frequencies *f* in Hz (parameters of the curves) and their fits by a sequence of tanh profiles (lines), the curves are shifted vertically for clarity. (**b**) The relative widths $p_j$ of individual tanh profiles. The inclined dashed lines denote the time dependences $t^\beta , \ \beta =0.3,0.5,0.7$. In panels (**c**–**e**) we plot the dependences of the growth rate *r* (**c**), the characteristic profile width $p_{20}$ (**d**) and the slope $\beta$ on the laser frequency *f*. In sample $f=2$ Hz the slope could not be determined.

Measured AB intensities *I*(*t*) are plotted in Fig. 1, panel (a). We used a sequence of tanh profiles according to Eq. (1) and simulated the AB intensity using relation:

2 $$\begin{aligned} I(t)={\mathrm {const}}. \left| \sum _{j=1}^N \theta _j(t) (-1)^j \right| ^2. \end{aligned}$$

From the fit to the experimental AB intensity *I*(*t*) we determined the relative widths $p_j$ and the characteristic times $t_j$ of individual profiles $\theta _j(t)$. In order to get a satisfactory high resolution it was necessary to include more than 50 tanh profiles. A reasonably robust fit was achieved only if we assumed that the growth rate *r* was constant during the deposition run, i.e. we assume $t_j={\mathrm {const.}}+jr$.

The results of the fitting are plotted in Fig. 1. Panel (a) shows the measured AB intensities (points) and their fits (lines) using Eq. (2). The correspondence of the measured and fitted curves is quite good except for the sample deposited at the laser frequency $f=2$ Hz, where the measured AB oscillations changed their period during the growth and the fit was a bit worse compared to the other samples. Panel (b) displays the time dependence of the relative widths $p_j$. The time dependence of the widths obeys the $t_j^\beta$ scaling except for the sample with $f=2$ Hz. In (c) we plot the dependence of the growth rate *r* on the laser frequency *f*. As expected, *r* increases linearly with *f*. Panels (d) and (e) display the relative width $p_{20}$ of the 20th profile and the slope $\beta$. Both of them decrease with increasing laser frequency.

**Figure 2:**
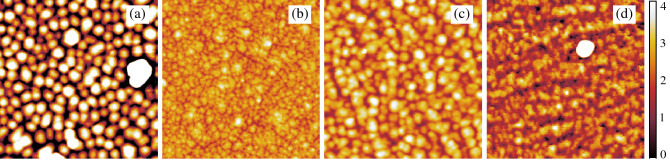
The AFM images of samples deposited at various laser frequencies: $f=1$ Hz (**a**), 2 Hz (**b**), 3 Hz (**c**), and 5 Hz (**d**). Lateral size of images is 1 $\upmu$m and the color bar denotes height in nm same for all images.

Figure 2 shows AFM images of samples deposited at various laser frequencies *f*. The AFM micrographs were measured at room temperature after the completion of about 140 layers. All deposited films are notably smooth—see the z scale bare of 4 nm. The RMS roughness measured from the AFM is 1.5 nm (1 Hz), 0.4 nm (2 Hz) and 0.6 nm (3 and 5 Hz) in a good agreement with the roughness determined by the X-ray specular reflectivity measured after the growth completion. Especially in the case of the sample deposited at the 1Hz laser frequency surface islands separated by narrow valleys can be distinguished, likely representing grains of LFO protected from coalescence. This effect was observed during growth of LFO on sapphire substrate with and without platinum interlayer^7^. The 3 and 5 Hz samples are influenced by a high density of steps on the substrate. To minimize effect of the granularity and the steps we will in further discussion focus only on the beginning of growth (first $\sim 20$ layers) with well defined layer-by-layer growth indicated by SXRD (see Fig. 1).

In the following section we discuss dependence of growth parameters from the SXRD oscillations on the laser frequency *f*. In principle, undetected structural or stoichiometric defects with formation dependent on *f* should be considered as a possible source of the dependency of the parameter $\beta$ on *f* as shown in Fig. 1e. We expect that amount of the defects sufficient for influencing the SXRD oscillations during the growth of the first layers would affect the morphology of much thicker layers observed by AFM ex-situ (Fig. 2). Because the observed grain size does not depend strongly on *f*, we will consider only idealized non-defected growth in the model.

### Monte Carlo simulations of the growth

In order to understand the effect of the laser frequency on morphology of the growing film we employed kMC simulations of the growth based on Bortz–Kalos–Lebowitz model^27^ in the limit of fast diffusion developed for effective simulations of PLD growth^26^. Four processes are implemented in the model: deposition, condensation, dissolution, and interlayer transport; condensation is considered to be a barrier-less process, dissolution and interlayer transport are thermally activated. The rate of dissolution is driven by the bonding energy to substrate ($E_S$) and to neighboring occupied cell ($E_N$ per bond). The Ehrlich-Schwoebel barrier $E_{SB}$ controls the rate of the interlayer transport. The rate of each process is defined as $R_X = R_0 \exp \left( - E_X / k_b T \right)$, where $E_X$ is an energy barrier of given process, $k_b$ Boltzmann constant and *T* temperature. The rates are only dependent on the ratio of $E_X$ and *T*, hence the exponent can be written as number $\varepsilon _X = E_X / k_b T$. The lattice model was developed for simulations of layer-by-layer growth on large domains over long times. A high efficiency of the model was achieved by approximating detailed atomic or molecular diffusion by means of 2D gas (the limit of fast diffusion) and by neglecting the chemical processes during the growth. The smallest unit in the simulation is the whole molecule. Further details of the model and simulations of interlayer transport can be found in our previous paper in Ref. ^26^.

The growth temperature and the laser frequency as parameters of the simulation were set the same as in the experimental settings, i.e. T = $850 ^{\circ }$C and *f* = 5 Hz. Several different sets of binding energies were tested in order to obtain suitable exponents $\varepsilon _X$ for which the measured and simulated AB intensities are in a close agreement. The most similar simulated AB intensity was chosen by comparison of rate constants of exponential decays of magnitudes of first few local maxima in both AB intensities. The first peak was not considered in order to reduce the influence of the substrate.

**Figure 3:**
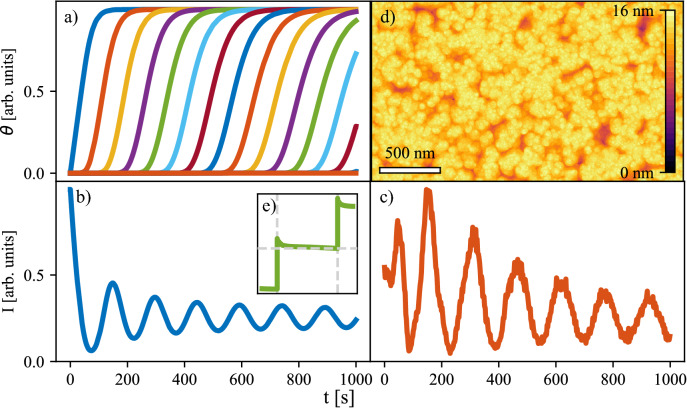
(**a**) The time dependencies of level coverages for first 1000 s, (**b**) the simulated anti-Bragg intensity, (**c**) the experimentally measured anti-Bragg intensity, and (**d**) the resulting morphology at the end of the simulation (8000 s). The inset (**e**) shows behaviour of level coverage after a pulse, the vertical dashed lines mark the depositions and the horizontal dashed line marks the level coverage at the last point before the pulse.

The results of the simulations using the best-fit parameters $\varepsilon _S = 13.5$ corresponding to dissolution of unit with no neighbors, $\varepsilon _{S+1N} = 20.5$ corresponding to dissolution of units with one neighbor and interlayer transport parameter $\varepsilon _{ESB} = 1.4$ are summarized in Fig. 3: evolution of level coverages (Fig. 3a), the AB intensity (Fig. 3b), experimentally measured AB intensity (Fig. 3c), and the resulting morphology (Fig. 3d). Besides the RMS roughness we calculate the correlation length as half-width of the main peak of the surface auto-correlation function. The correlation length roughly corresponds to the mean particle size.

The profiles are shaped approximately like hyperbolic tangents. The slopes of the profiles decrease, which is linked with decreasing magnitude of oscillation of the AB intensity (Fig. 3b via Eq. (2)). Figure 3e shows the evolution of selected level coverage between two deposition pulses after 300 s of growth. There is a visible decrease in the level coverage right after the pulse which is followed by a slower steady decrease lasting the whole period between two pulses. This effect is caused by dissolution of unstable parts of the layer in combination with transport of molecules from higher to lower level^20^.

**Figure 4:**
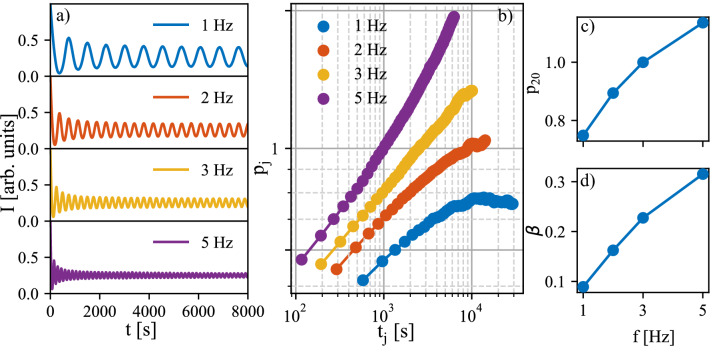
(**a**) Simulated AB intensities for simulations with different repetition frequencies *f* = 1, 2, 3, and 5 Hz. (**b**) The relative widths of individual level coverages fitted by tanh profiles. (**c**) The characteristic profile width $p_{20}$ and (d) the evolution of $\beta$ with frequency.

Influence of the laser repetition frequency on the growth simulated using the barriers found for $f= 5$ Hz is presented in Fig. 4. The simulated AB intensities (Fig. 4a), the evolution of widths of individual profiles (Fig. 4b), the evolution of relative width of 20th profile (Fig. 4c), and the scaling exponent $\beta$ (Fig. 4d) can be compared to the results extracted from AB data in Fig. 1. The scaling exponent was obtained by fitting each level coverage by hyperbolic tangent (cf. Eq. 1) in the same way as in the case of the experimental data. Evidently, the dependence on frequency is opposite in the simulations: $p_{20}$ and $\beta$ values are increasing with increasing laser repetition frequency. Further simulations with repetition frequencies up to 1000 Hz were made and it was found that the increase of $\beta$ slows down for higher repetition frequencies and the value saturates at $\sim 0.534$. In the experimental data the $\beta$ parameter increases up to 1 (Fig. 1). The difference might be caused by an effect which is not considered in the model, for example the original state of the substrate is perfectly flat in the simulations. In the experiment there might be defects on the substrate influencing the growth. Nevertheless, the exact value of the $\beta$ is not as important as the trend of the change of the $\beta$ parameter with frequency. The simulated dependence corresponds to the rougher interfaces in case of higher frequencies, which is expected due to shorter time for relaxation between the pulses mentioned above (see Fig. 3e).

The impinging laser ablated particles in PLD may significantly increase the surface temperature. In Ref. ^28^ the authors used a 2D heat transfer model to calculate temperature increase during PLD growth. In this work, the energy of the arriving plume of particles deposited to the substrate was calculated assuming an incident energy flux density in the form of a cylindrically symmetric gaussian distribution, and using a heat conduction equation. From the simulations they determined the time and space distribution of the local increase of the substrate temperature, showing that the maximum temperature increase at the surface can exceed 50 K, depending on the mean energy and flux density of the impinging particles.

We repeated this approach in a simplified form assuming a flux density of the arriving particles on the substrate surface in the form of very short pulses homogeneous in space; the value of the amount of particles deposited in one pulse was deduced from the known growth rate. We calculated the temperature increase as function of time after the pulse arrival and depth in the substrate, as well as the temperature averaged over the pulse period. The substrate temperature substantially depends on the kinetic energy of incoming particles and optical emissivity of LuFeO$_3$ at the growth temperature. Since the exact values of these parameters are not know, the results of the calculations can be interpreted only as a qualitative demonstration of the existence of an temperature increase and a very rough estimate of its value. From the calculation it follows that the temperature increase averaged over the laser period 1/f between the pulses is proportional to the mean particle energy and the laser frequency, and it slowly increases during the deposition. For instance, for the mean energy of incoming particles of 2 eV, frequency 10 Hz, and the pulse length of 1 $\upmu$s, the increase of the substrate surface temperature between the pulses No 50 and 51 averaged over the period between the pulses compared with original substrate temperature was approximately 220 K.

**Figure 5:**
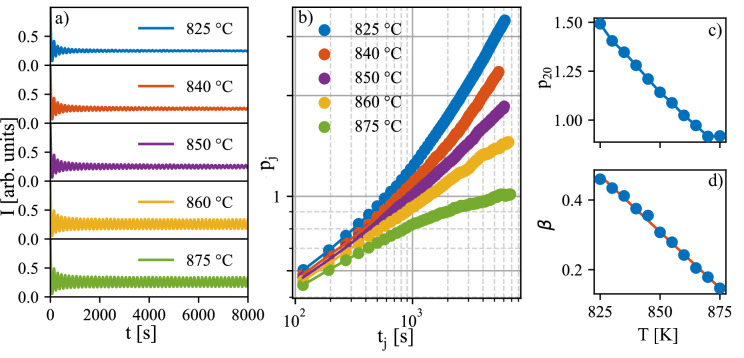
(**a**) Simulated AB intensities for simulations with increasing temperature for chosen temperatures T = 825, 840, 850, 860, and 875 $^{\circ }$C. (**b**) The relative widths of individual level coverages fitted by tanh profile. (**c**) The characteristic profile width $p_{20}$ and (**d**) the evolution of $\beta$ with temperature.

In order to test if the surface temperature increase could be responsible for the experimentally obtained dependence on the laser repetition frequency (Fig. 1), we simulated the influence of the growth temperature on growth characteristics (Fig. 5). Evidently, the 20th profile width and scaling exponent $\beta$ decrease with temperature similarly to the measured dependence on the repetition frequency. Because the simulated effects of temperature and the repetition frequency are opposite (see Figs. 4 and 5), the temperature increase by the impinging particles would have to compensate and overcome the effect of the repetition frequency.

**Figure 6:**
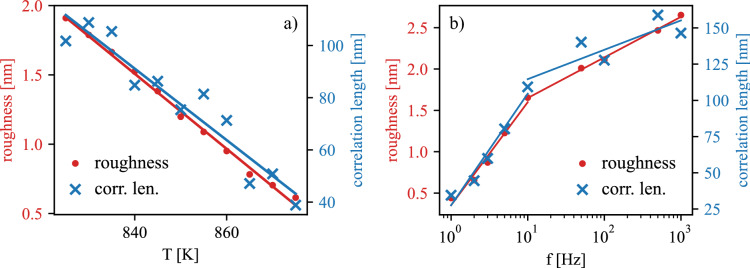
Dependency of simulated roughness and correlation length on temperature (**a**) and deposition frequency (**b**). The lines are guides for the eye.

In order to gain more information about the simulated morphologies, the dependency of surface roughness and mean correlation length on the substrate temperature and the repetition frequency are shown in Fig. 6a and b, respectively. The figure shows that both the roughness and the mean correlation length scale together and thus in following we discuss only the surface roughness. The roughness decreases with temperature, which is caused by increasing rate of dissolution of units and their subsequent jump down to lower layer, since both processes are thermally activated. The increased roughness with the repetition frequency can be explained using the inset in Fig. 3e. The slow but observable interlayer transport in the period between the pulses is caused by dissolution of weakly bounded molecules (either with zero or one neighbor) and their subsequent jump to the lower level. As the deposition frequency rises the subsequent depositions are divided by smaller time margin and thus the the system is further away from the equilibrium (flat compact 2D film with only one level open) when the next deposition occurs.

**Figure 7:**
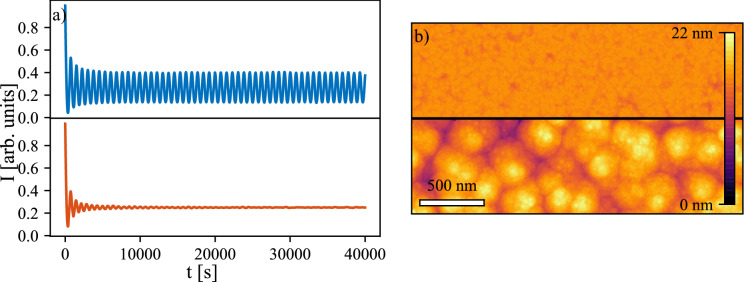
(**a**) The comparison of AB intensities and (**b**) morphologies obtained for nominal temperature obtained from 5 Hz simulations (top) and for corrected temperature obtained from 1 Hz simulations (bottom).

The simulations allow to find the difference between the effective temperatures corresponding to 1 Hz and 5 Hz. The non-dimensional exponent $\varepsilon _X$ is only a function of temperature if it is assumed that there is no process which would modify the energy barrier when deposition frequency changes. Hence, in order to obtain a different $\varepsilon _X$ for different deposition frequencies, the effective temperature of growth must change. Comparing the best fit values of $\varepsilon _X$ for samples obtained using 1 Hz and 5 Hz deposition frequencies it can be found that the effective surface temperature increases by about $7\,\%$. Figure 7 shows the AB intensities (Fig. 7a) and morphologies (Fig. 7b) obtained from the simulation of 1 Hz laser frequency using the $\varepsilon$ found by fitting the 5 Hz, i.e. without the temperature correction (top panels), and 1 Hz (bottom panels) AB data. Both the AB intensities and morphologies show that the 7 % growth temperature change is significant enough to majorly influence the growth. In the higher temperature case the growth is very close to layer-by-layer while in the other case distinct islands can be observed.

## Discussion

The kMC model provides an explanation of the decreasing exponent $\beta$ with increasing repetition frequency. It was found that the increase is not directly caused by the repetition frequency change itself but it is due to the temperature increase of the first few layers of the substrate, which is frequency dependent.

In the experimental setup the substrate temperature is kept constant during the deposition by a thermocouple which is placed on the back side of the substrate. This configuration is able to keep the nominal temperature constant up to small fluctuations. However, the temperature on the front side where the growth occurs can be different and varies quickly during the deposition. The effective temperature used in the simulations is a temperature at which the number of processes occurring between two pulses is similar to the number of processes occurring in a growth with the actual variable temperature.

The increasing temperature significantly increases the rates of processes responsible for smoothing the surface. The obtained temperature increase by $\sim$ 7 % leads to $\sim$ 9.5 % increase of the rate of jump down to lower layer, 2.5 times increase in rate of dissolution of condensated units with no neighbors and 3.75 times increase for units with one neighbor.

The existence of the temperature difference after deposition between front and back is in agreement with calculation performed by Xu (Ref. ^28^) and our own simpler 1D model where the temperature as function of distance from the surface was found. There is a steep temperature gradient which is caused by low thermal conductivity of the YSZ substrate. Our results demonstrate that the difference between nominal and effective surface temperatures needs to be taken into account during PLD growth, especially for substrates with very low thermal conductivity because the effect gets stronger with decreasing thermal conductivity ^28^.

Besides the repetition frequency, other laser parameters, such as laser fluence are known to affect the PLD growth^29–31^. Significant variation of the fluence can modify the resultant stoichiometry and crystal structure. However, in case of moderate variation of the fluence we expect the similar effect as that of the repetition frequency—higher flux of arriving material will cause higher increase of the surface temperature.

## Conclusion

We have presented measured data of LuFeO$_3$ growth using pulsed laser deposition. The scaling exponent $\beta$ was calculated from the SXRD data obtained during the deposition for various deposition frequencies. It was found that this exponent is decreasing with increasing deposition frequency.

Using the SXRD data for 5 Hz and 1 Hz deposition frequencies the parameters for a kinetic Monte Carlo model were determined. It was found that the simulated behaviour of the scaling exponent $\beta$ is opposite to that observed experimentally. After a further study, it was found that the behaviour of the exponent $\beta$ can be explained by increasing temperature of few topmost layers during the deposition. The increasing temperature due to impinging flux of ablated particles is more significant for higher deposition frequencies and should be taken in account, especially in case of PLD growth of materials with low thermal conductivity.

## Methods

Hexagonal LuFeO$_3$ layers were grown by the PLD method at the growth temperature of $T_{\mathrm {g}}=850^\circ C$ on yttria-stabilized zirconia (YSZ) (111)-oriented substrates annealed for 2 hours in a furnace in oxygen environment. The layers were deposited using the laser fluence of 1.5 J/cm$^2$, the laser wavelength was 266 nm, the laser spot size on the target was 0.075 cm $\times$ 0.125 cm, and the target-substrate distance 60 mm. We performed four growth runs with Qsmart 850 YaG laser from Quantal company with a pulse duration of 5 ns and laser frequencies $f = 1,2,3,5$ Hz.

The PLD chamber is installed on a multipurpose heavy-duty X-ray diffractometer on the NANO beamlime at synchrotron KARA (KIT, Karlsruhe, Germany)^32–34^. The intensity of the LuFeO$_3$ 0003 quasiforbidden diffraction spot (AB intensity) was acquired in situ during the deposition, using an one-dimensional (1D) Mythen detector (Dectris) having 1280 channels with the channel size of 50 $\upmu$m, the acquisition time was 1 s. The detector was placed 1118 mm behind the sample giving an angular resolution of 0.00257 degree per channel. The X-ray beam with the energy of 15 keV had the beam size in FWHM of 250 $\upmu$m (horizontal)$\times$80 $\upmu$m (vertical).
